# Exploring long-term retention and reactivation of micropollutant biodegradation capacity

**DOI:** 10.1007/s11356-024-34186-w

**Published:** 2024-07-10

**Authors:** Rita H. R. Branco, Roel J. W. Meulepas, Huub H. M. Rijnaarts, Nora B. Sutton

**Affiliations:** 1 Environmental Technology, Wageningen University & Research, P.O. Box 47, 6700 AA Wageningen, the Netherlands; 2 Wetsus, European Centre of Excellence for Sustainable Water Technology, P.O. Box 1113, 8900 CC Leeuwarden, the Netherlands

**Keywords:** Micropollutant biodegradation capacity, Dissolved organic carbon, Micropollutant concentration, Biostimulation, Micropollutant biodegradation reactivation, Acetate

## Abstract

The factors limiting micropollutant biodegradation in the environment and how to stimulate this process have often been investigated. However, little information is available on the capacity of microbial communities to retain micropollutant biodegradation capacity in the absence of micropollutants or to reactivate micropollutant biodegradation in systems with fluctuating micropollutant concentrations. This study investigated how a period of 2 months without the addition of micropollutants and other organic carbon affected micropollutant biodegradation by a micropollutant-degrading microbial community. Stimulation of micropollutant biodegradation was performed by adding different types of dissolved organic carbon (DOC)—extracted from natural sources and acetate—increasing 10 × the micropollutant concentration, and inoculating with activated sludge. The results show that the capacity to biodegrade 3 micropollutants was permanently lost. However, the biodegradation activity of 2,4-D, antipyrine, chloridazon, and its metabolites restarted when these micropollutants were re-added to the community. Threshold concentrations similar to those obtained before the period of no substrate addition were achieved, but biodegradation rates were lower for some compounds. Through the addition of high acetate concentrations (108 mg-C/L), gabapentin biodegradation activity was regained, but 2,4-D biodegradation capacity was lost. An increase of bentazon concentration from 50 to 500 µg/L was necessary for biodegradation to be reactivated. These results provide initial insights into the longevity of micropollutant biodegradation capacity in the absence of the substance and strategies for reactivating micropollutant biodegrading communities.

**Graphical abstract:**

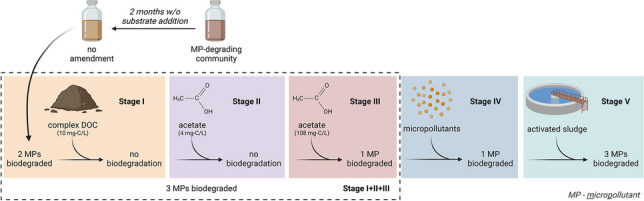

**Supplementary Information:**

The online version contains supplementary material available at 10.1007/s11356-024-34186-w.

## Introduction

Micropollutants, such as pharmaceuticals and personal care products (PPCPs), pesticides, and industrial chemicals, are increasingly found in drinking water sources (Benner et al. 2013). For this reason, the European Union (EU) created multiple directives setting a threshold concentration for micropollutants in these sources, such as surface and groundwater (European Commission 2006, 2008). Although these compounds are typically found in the range of picograms per liter (pg/L) to micrograms per liter (µg/L), they are often above the levels set by the EU (Benner et al. 2013). Micropollutants can reach waterbodies via diffuse and point sources. Diffuse sources include, for example, pesticides applied in agricultural fields leaching from soil to groundwater. Wastewater treatment plant (WWTP) effluent discharges and landfills are point sources of contamination with PPCPs and industrial substances (Mukhopadhyay et al. 2022). Microbial communities at the source of contamination such as soil and activated sludge (Li et al. 2013b; Bertelkamp et al. 2015; Kowalska et al. 2019; Kennes-Veiga et al. 2021a, 2021b; Brucha et al. 2021) as well as in the drinking water sources (Liu et al. 2013; Burke et al. 2017; Luo et al. 2019; Coll et al. 2020) can biodegrade micropollutants.

The persistence of micropollutants in surface and groundwater indicates that micropollutant biodegradation in these different environments (i.e., natural attenuation) occurs to a low extent or at a slow rate. For this reason, extensive research has been performed to understand how factors like adaptation time (Suarez et al. 2010; Hoppe-Jones et al. 2012; Bertelkamp et al. 2016; Poursat et al. 2019a, b) and, to a lesser degree, redox condition (Burke et al. 2017; Schmidt et al. 2017; de Wilt et al. 2018; Branco et al. 2023) affect micropollutant biodegradation. Additionally, many authors focused on stimulating this process via amendment with, for example, dissolved organic carbon (DOC) (Lim et al. 2008; Maeng et al. 2011; Drewes et al. 2014; Li et al. 2014; Alidina et al. 2014; He et al. 2018; Luo et al. 2019). When biodegradation is successfully improved in the environment, the micropollutant is eventually depleted, and if biostimulation is performed, the addition of the stimulating compound will be ceased. Consequently, the microbial community responsible for the micropollutant removal will no longer be exposed to the micropollutant or the stimulant. It is known that fluctuations in the environment, such as the presence of nutrients and substrates, result in changes in microbial community composition. If the time scale of the fluctuation is much slower than the response of the microbial community to it, permanent community composition changes can occur (Nguyen et al. 2021). Therefore, it is possible that exposure to a prolonged period without micropollutants and other organic carbon sources results in a decrease in the concentration of micropollutant degraders and, eventually, a complete loss of micropollutant biodegradation capacity.

Since re-contamination of the system with the micropollutant can occur, maintaining an established micropollutant-degrading community during a long period of no micropollutant exposure or other organic carbon addition is of great importance. However, little information is available on this topic, and the present work is, therefore, a first attempt to understand the capacity of a microbial community to restart micropollutant biodegradation once micropollutants are again available. Such understanding is valuable not only in the case of re-contamination of a previously bioremediated system with micropollutants (e.g., via biostimulation or bioaugmentation), but also for systems removing micropollutants via biodegradation and in which the influent composition, including micropollutants and other organic carbon sources, fluctuates—such as WWTPs (Vieno et al. 2005; Kot-Wasik et al. 2016; Di Marcantonio et al. 2020b)—constructed wetlands (Matamoros et al. 2008; Maurice et al. 2022), and biological activated carbon and rapid sand filters (Ross et al. 2019; Di Marcantonio et al. 2020a; Wang et al. 2021). Furthermore, it is also essential to understand which strategies could be applied to stimulate the reactivation of micropollutant biodegradation. For example, amendment with electron donors such as DOC and ammonium could be used to stimulate heterotrophic and autotrophic co-metabolic micropollutant biodegradation, respectively (He et al. 2018; Su et al. 2021; Aldas-Vargas et al. 2021). DOC could also be used by microorganisms as a source of energy and building blocks for the induction of catabolic genes for micropollutant degradation (Egli 2010; Hübner et al. 2022). Finally, a well-known reason for micropollutant persistence in the environment is that below certain low concentrations, micropollutant biodegradation is not energetically favorable (i.e., the energy gained is lower than the energy required for degradation), which causes micropollutant biodegradation to stop (Kundu et al. 2019; Wirsching et al. 2020). Hence, it is possible that reactivation of micropollutant biodegradation does not occur until micropollutants accumulate above those concentrations.

This study aimed to understand to what extent a microbial community adapted to multiple micropollutants can maintain its micropollutant degradation capacity after a period of no substrate addition (i.e., micropollutants and other organic carbon sources) and how any loss in biodegradation activity can be mitigated. For this, a mixture of two microbial communities previously exposed to micropollutants and presenting the combined ability to biodegrade 12 different micropollutants under aerobic conditions was selected. After 2 months without further micropollutant and other organic carbon addition, a series of five sequential amendments was performed to stimulate micropollutant biodegradation: (i) addition of complex DOC with different biodegradability, (ii) addition of simple DOC (acetate) in low concentration, (iii) addition of simple DOC (acetate) in high concentration, (iv) addition of micropollutants to increase the concentration 10 × , and (iv) inoculation with a different microbial community (activated sludge). The results obtained contribute to understanding the capacity of a microbial community to reactivate micropollutant biodegradation once a remediated environment is again contaminated with micropollutants.

## Materials and methods

### Micropollutants

The present study evaluated the removal of 16 micropollutants: 6 herbicides (mecoprop (MCPP), 2,4-dichlorophenoxyacetic acid (2,4-D), bentazon (BTZ), dichlobenil (DCB), chloridazon (CLZ), and metolachlor (MET)), 5 herbicide metabolites (2,6-dichlorobenzamide (BAM), chloridazon-desphenyl (CLZ DP), chloridazon-methyl-desphenyl (CLZ MDP), metolachlor ESA (MET ESA), and metolachlor OA (MET OA)), 3 pharmaceuticals (gabapentin (GAB), carbamazepine (CBZ), and antipyrine (ANP)), 1 artificial sweetener (acesulfame K (ACK)), and 1 industrial substance (1H-benzotriazole (1H-BTR)). The compounds were selected because they are frequently detected in Dutch groundwater (van Loon et al. 2020; Wageningen University and Research 2022) (concentration and frequency of detection in Table S1, Supplementary material). Additionally, they have a wide variety of physico-chemical properties (Supplementary information, Table S2). Two micropollutant stock solutions were prepared in Milli-Q water, one with 1.5 mg/L and another with 27 mg/L of each micropollutant.

### Microbial community sources

In one of our previous studies, soil from agricultural fields and sediment from the surrounding ditch were spiked, in batch bottles and under aerobic conditions, with 500 µg/L of each of the above-mentioned micropollutants (1st spike) and, 100 days later, re-spiked with the same amount (2nd spike) (Branco et al. 2023). The soil-derived community showed 2,4-D, MCPP, CLZ, MET, BTZ, ANP, CBZ, GAB, and 1H-BTR degradation activity during the 1st spike and 2,4-D, MCPP, CLZ, MET, ANP, and 1H-BTR degradation activity during the 2nd spike. While the ditch-derived community was able to degrade 2,4-D, ANP, GAB, and 1H-BTR during the 1st spike and CLZ, CLZ DP, CLZ MDP, and ANP during the 2nd spike. The communities obtained at the end of this experiment were further incubated for 2 months at 20 °C, 120 rpm, and in the dark without any further micropollutant and other organic carbon addition. The resulting microbial communities were used as inoculum in the present study. The initial inoculum used in this study consisted of a mixture of the resulting soil- and ditch-derived communities in a ratio of 1:1 (v:v).

Activated sludge collected from an aerobic tank of the municipal WWTP in Bath (Waterboard Brabantse Delta, the Netherlands) was also used as inoculum. A pre-treatment to allow the oxidation of all the easily degradable carbon to CO_2_ and the conversion of ammonium to nitrate was done as follows: 70 g of sludge (wet weight 57.34 g-TSS/L and 43.67 g-VSS/L) were diluted in 1 L tap water. The diluted sludge was then incubated overnight in a 1-L working volume reactor and thermoregulated at 25 °C and under 150 rpm agitation. During the incubation, the sludge was aerated with water-saturated air at the flow rate of 1 L/min.

### Dissolved organic carbon

Throughout this study, five different DOC types were tested: (i) GC (green compost), extracted from green compost with a composition of 50% screened wood, 25% grass litter, and 25% leaf litter (Van Iersel Compost, the Netherlands); (ii) GFTC (GFT compost), extracted from GFT compost produced from vegetable, garden, and fruit waste (Attero, the Netherlands); (iii) HA (humic acids), extracted from humic acids concentrated by an anion exchange resin from natural groundwater (Vitens, the Netherlands); (iv) LH (liquid humus), extracted from liquid humus obtained from leonardite (Soiltech, the Netherlands); and (v) acetate, sodium acetate ≥ 99.0%, AnalaR® NORMAPUR® (VWR Chemicals). GC, GFTC, LH, and HA are complex DOC types, with GC and GFTC being more labile than LH and HA (see Table S3 for DOC type composition). On the other hand, acetate is a simple and easily biodegradable DOC type.

DOC extraction was required for GC and GFTC. For this, Milli-Q water was mixed with the DOC source in a 4:1 ratio (w:w). The obtained solution was shaken overnight at 120 rpm. The obtained suspensions were then centrifuged twice for 15 min at 17,700 rcf; the pellet was discarded after each centrifugation. Finally, the supernatant solution was filtered through a 0.45-μm membrane filter (Whatman-ME 25/21 ST) using a vacuum filtration system. To ensure that no particles were present in LH and HA, the extraction procedure was also applied to the source of these DOC types. Prior to use, the DOC extracts were diluted to a concentration of 150 mg-C/L and stored at 4 °C.

Two sodium acetate stock solutions were prepared in Milli-Q water, one containing 1.4 g/L sodium acetate (i.e., 0.41 g-C/L) and another containing 26.6 g/L sodium acetate (i.e., 7.8 g-C/L).

### Sand

The sand was used as a solid matrix to support the growth of biofilm colonies. The sand used in the experiment was kindly supplied by Deltares (the Netherlands) and consisted of silica particles with a uniform size of 200 µm. The sand was autoclaved before use.

### Sodium azide

To exclude micropollutant removal due to abiotic processes (i.e., adsorption, dissociation, and oxidation), O_2_ uptake by microorganisms and, consequently, microbial activity were inhibited by adding sodium azide (NaN_3_) in abiotic batches. A stock solution of 6.6 g/L NaN_3_ was prepared in Milli-Q water.

### Experiment design

Batch experiments were performed to investigate the limitations to the degradation of the selected micropollutants under aerobic conditions. The experiments were performed in batch bottles (250 mL) sealed with Viton® rubber stoppers. The bottles were incubated at 20 °C, 120 rpm, and in the dark to avoid photodegradation of the micropollutants. The bottles were filled with 120 mL of media containing approximately 280 mg-NH_4_^+^/L, with pH 7.0, and prepared with Milli-Q water (see Table S4 for the initial media composition), 15 g of sand, and 5 mL of the micropollutant stock solution with 1.5 mg/L.

The bottles were divided into four different types of batches: no-DOC (batches inoculated with 15 mL of soil/ditch but with no external DOC addition), biotic (batches inoculated with 15 mL of soil/ditch and to which DOC was added from a stock solution to a final concentration of 10 mg-C/L), blank (batches without inoculum and to which DOC was added from a stock solution to a final concentration of 10 mg-C/L), and abiotic (batches inoculated with 15 mL of soil/ditch and to which DOC was added from a stock solution to a final concentration of 10 mg-C/L, as well as 3 mL of sodium azide stock solution).

The headspace of the bottles was filled with compressed air (N_2_/O_2_ 80%/20%, 1 bar over-pressure). The first stage (I) of the experiments was started by adding 5 mL of the micropollutant stock solution, yielding a final concentration of 50 µg/L for each micropollutant. Table 1 shows an overview of the composition of each batch at the beginning of Stage I, including microbial community source, DOC type, and micropollutant concentration. All batches were performed in triplicate.

**Table 1:** Overview of the composition of each batch type at the beginning of Stage I

Batch type	Microbial community		DOC^a^		Milli-Q water^b^	NaN_3_^c^	Micro-pollutants^d^	Media	Sand
	Source	Volume	Type	Volume					
No-DOC	Soil/ditch^e^	15 mL	–	–	10 mL	–	5 mL	120 mL	15 g
Biotic	Soil/ditch^e^	15 mL	GC	10 mL	–	–	5 mL	120 mL	15 g
			GFTC	10 mL					
			LH	10 mL					
			HA	10 mL					
Blank	–	–	GC	10 mL	15 mL	–	5 mL	120 mL	15 g
			GFTC	10 mL					
			LH	10 mL					
			HA	10 mL					
Abiotic	Soil/Ditch^e^	15 mL	GC	10 mL	–	3 mL	5 mL	120 mL	15 g
			GFTC	10 mL					
			LH	10 mL					
			HA	10 mL					

^a^Final concentration of about 10 mg-C/L

^b^Added to reach similar working volume when inoculum or DOC were not added

^c^Final concentration 130 mg/L

^d^1.5 mg/L stock solution, final concentration 50 µg/L

^e^Ratio 1:1 (v:v)

In order to stimulate micropollutant biodegradation, a series of four sequential amendments were performed, starting on day 125, to the batches (each corresponding to a different stage—II to V). These amendments are listed in Table 2, including the day of amendment, solutions added, and to which batches. The experiments ended after a total of 349 days.

**Table 2:** Sequential amendments to the batches (Stages II, III, IV, and V)

Stage	Day	Batch	Modifications	Solutions added
II	125	All	Addition of 4 mg-C/L of acetate	1 mL sodium acetate stock solution (1.4 g/L)
III	160	All	Addition of 108 mg-C/L of acetate	1 mL sodium acetate stock solution (26.6 g/L)
IV	244	All	Concentration of each micropollutant increased to 500 µg/L^a^	1 mL micropollutant stock solution (27 mg/L)
V	321^b^	No-DOC, all blank, abiotic GFTC	Inoculation of new microorganisms and renewal of nutrients	2 mL activated sludge community, 80 mL fresh media^c^

^a^To stimulate metabolic activity and natural selection

^b^Changes performed to 2 bottles of each of the batches

^c^Addition of media diluted the concentration of each micropollutant to about 160 µg/L

### Chemical analysis

Liquid and gas samples (2.5 and 3.0 mL, respectively) were collected throughout the experiments for chemical analysis. Detection limits for all compounds and the batch types in which the compounds were analyzed can be found in Table S5 and Table S6, respectively. For micropollutant analysis, liquid samples were centrifuged twice for 10 min at 20,000 rcf, and the pellet was discarded after each centrifugation. For the other analysis, liquid samples were centrifuged for 10 min at 20,000 rcf, and the resulting supernatant was filtered (0.45 µm PTFE syringe filter). Reported values are the average and standard deviation of the replicates. An Rmarkdown workflow of the chemical data analysis is accessible from https://github.com/rbranco01/micropollutant_biodegradation_limitations.

#### Micropollutants

Micropollutant concentrations were determined with a liquid chromatograph-mass spectrometer (Agilent 6420 LC–MS/MS) with a selective electrospray, triple quad LC–MS/MS in multiple reaction monitoring (MRM) transitions. An Agilent ZORBAX Eclipse Plus C18 RRHD column (1.8 µm, 50 × 2.1 mm) equipped with a guard column (UHPLC guard ZORBAX Eclipse Plus C18, 1.8 µm, 2.1 × 5 mm) was used for the analysis. The mobile phase consisted of a mixture of eluent A (neutral buffer of 10 mM ammonia, 10.4 mM formic acid, and 0.04 mM oxalic acid) and eluent B (acetonitrile) with a flow rate of 0.25 mL/min. Prior to analysis, all samples (1 mL) were spiked with a matrix modifier (50 µL) and an internal standard (50 µL). Matrix modifier solution was prepared by adding 50 mL MQ water, 8 mL formic acid, 3.3 mL 5 M ammonia, and 1 mL 1 M oxalic acid. Samples were also spiked with a micropollutant standard (50 µL), and recoveries were determined to account for matrix effects. The composition of the standards used can be found in Table S7. The sample injection volume was 3 μL. The results were acquired and analyzed using Agilent Mass Hunter Quant software. A micropollutant was considered to have been biodegraded if removal efficiency was ≥ 30% and did not occur in the abiotic batches.

#### Dissolved organic carbon

DOC fractions were analyzed with liquid chromatography-organic carbon detection (LC-OCD). LC-OCD separates DOC into fractions according to their molecular weight (Table S8)—biopolymers, humics, low molecular weight neutrals, low molecular weight acids, and hydrophobic organic carbon. Samples were analyzed using LC-OCD model 8 (DOC-Labor) with a built-in Siemens Ultramat 6E Non-Dispersive Infra-Red detector, coupled with an Agilent 1260 Infinity organic nitrogen detector (UV 220 nm) and a UV detector (254 nm). A Toyopearl HW-50S column (30 µm, 20 × 250 mm) was used for the analysis, and the mobile phase consisted of phosphate buffer (28 mmol, pH 6.6).

Samples were analyzed for acetate with a Metrohm Compact IC Flex 930 with a chromatographic column (Phenomenex Synergi 4u Hydro-RP 80A), a guard column (Metrohm Metrosep Organic Acids Guard/4.6), and a conductivity detector.

#### Ions

Samples were then analyzed for NH_4_^+^ with a Metrohm Compact IC Flex 930 with a cation column (Metrohm Metrosep C 4–150/4.0), a guard column (Metrohm Metrosep RP 2 Guard/3.5), and a conductivity detector. Samples were also analyzed for anions NO_3_^−^ with a Metrohm 930 Compact IC Flex with an anion column (Metrohm Metrosep A Supp 5–150/4.0), a guard column (Metrohm Metrosep A Supp 4/5 Guard), and a conductivity detector.

#### Headspace analysis

Gas samples were analyzed for O_2_, CO_2_, and CH_4_ by gas chromatography (Varian CP-4900 Micro-GC) with a Mol Sieve 5 Å PLOT (MS5) column (10 m × 0.53 mm, 30 µm, fused silica, aluminosilicate phase), a PoraPLOT U (PPU) column (10 m × 0.53 mm, 20 µm), and a thermal conductivity detector. The O_2_ and CO_2_ percentages were recalculated to millimole (mmol) using the ideal gas law and headspace volume and pressure.

## Results and discussion

### Initial micropollutant biodegradation activity

**Fig. 1:**
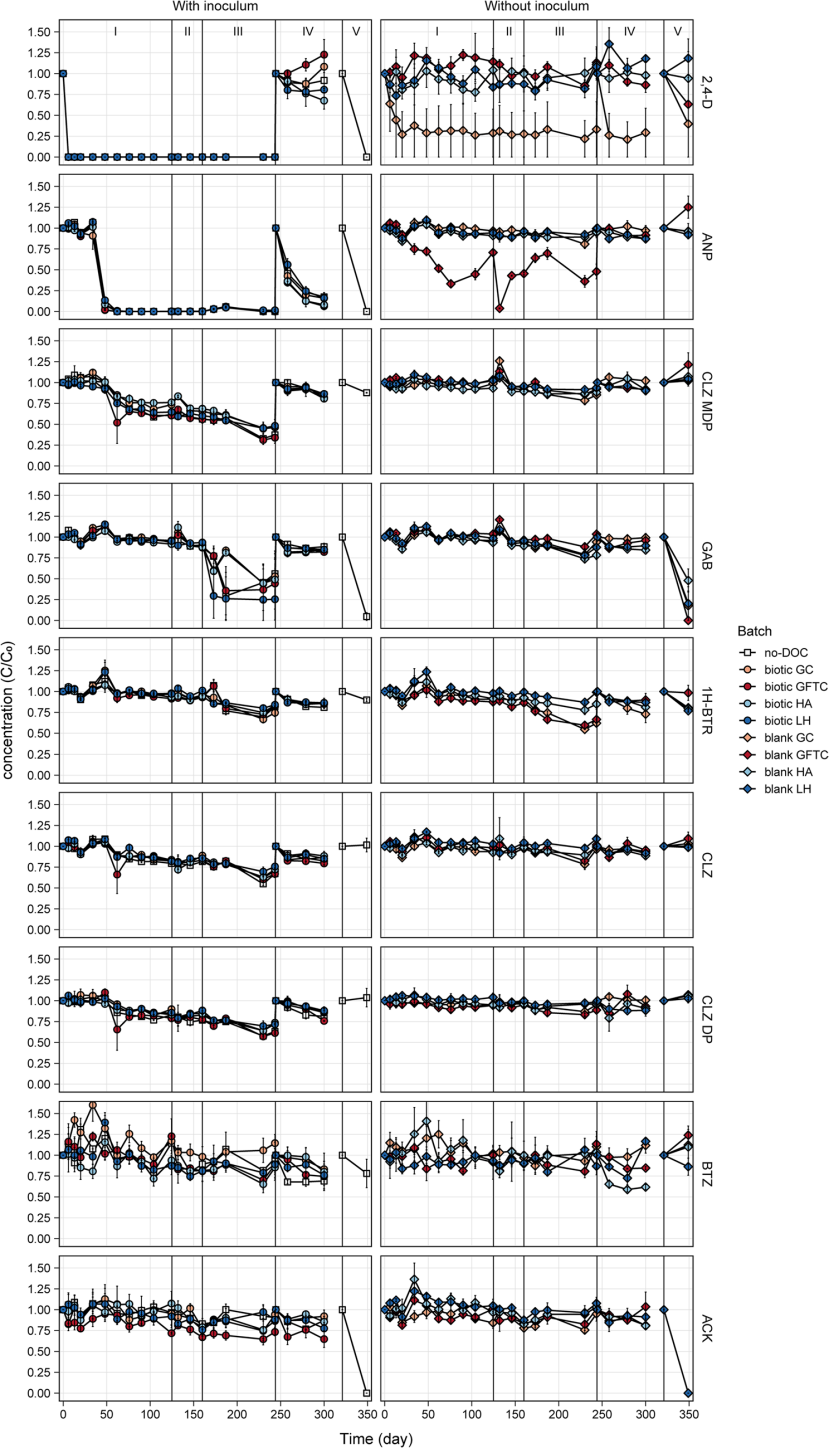
Micropollutant consumption for the no-DOC, biotic, and blank batches across the different stages

The extent to which biodegradation activity was retained was tested during Stage I. No amendment was performed to the no-DOC batches (i.e., control batches). Therefore, the biodegradation activity observed for these batches in the first 125 days of the experiment shows the biodegradation capacity retained by the microbial community which could be immediately reactivated when micropollutants are present again at low concentrations without a biostimulation strategy being applied. Biodegradation of 2,4-D, antipyrine (ANP), and chloridazon-methyl-desphenyl (CLZ MDP) was observed in the no-DOC batches during Stage I (Fig. 1). Biodegradation trends differed for each compound. 2,4-D biodegradation did not show a lag phase, and full biodegradation (i.e., below the detection limit; assessing if micropollutants are fully mineralized was not part of this study) was achieved by day 6. A lag phase of about 34 days was observed for ANP and CLZ MDP. Most ANP biodegradation occurred between days 34 and 48, and full biodegradation was observed by day 62. On the other hand, CLZ MDP was biodegraded at a lower rate, and a removal efficiency of 38.2 ± 8.7% was achieved by the end of Stage I (day 125). The much faster 2,4-D biodegradation was likely due to the presence of a higher number of microorganisms able to degrade this micropollutant in the inoculum. In fact, the *tfdA* gene, which encodes for the enzyme catalyzing the first step of 2,4-D biodegradation, is widespread in soil even in soil microbial communities not actively biodegrading this micropollutant (Hogan et al. 1997; Kim et al. 2013; White et al. 2022). On the other hand, ANP and CLZ MDP biodegradation in this environment is much less frequently reported and understood.

The capacity to biodegrade 2,4-D, ANP, and CLZ MDP had already been demonstrated by the soil and ditch microbial communities during the 2nd spike of our previous study (Branco et al. 2023). Hence, microorganisms with 2,4-D, ANP, and CLZ MDP biodegradation capacity were able to survive a period of 2 months without additional organic carbon sources. Furthermore, a slight increase in the concentration of these micropollutants was enough for their biodegradation to be reactivated, and no additional carbon substrate was necessary. Although removal of CLZ MD was much slower than previously observed for these microbial communities, the threshold concentrations reached for 2,4-D, ANP, and CLZ MDP in the present study are comparable with the ones observed at the end of the 2nd spike of our previous experiment (Table 3). Nevertheless, the ditch/soil communities had shown the ability to degrade the following 8 other micropollutants: gabapentin, 1H-benzotriazol, chloridazon, chloridazon-desphenyl, bentazon, MCPP, metolachlor, and carbamazepine (Branco et al. 2023), which were not removed in the no-DOC batches during Stage I. Different biostimulation strategies were, therefore, explored with the goal of reactivating the biodegradation of those micropollutants.

**Table 3:** Comparison between the micropollutant concentration at the end of 1st and 2nd spikes of our previous study and at the end of Stages III and IV of the present study

Inoc	Replicate	Concentration (µg/L)							Ref
		2,4-D	ANP	CLZ MDP	GAB	CLZ	CLZ DP	BTZ	
1st spike—day 100^a^									Branco et al. (2023)
Ditch	A	0.00	190	nd	331	nd	nd	nd	
	B	0.00	82.7	nd	248	nd	nd	nd	
Soil	A	0.00	18.5	nd	336	0.00	nd	258	
	B	0.00	23.6	nd	349	0.00	nd	282	
2nd spike—day 175^b^									
Ditch	A	nd	nd	nd	nd	nd	nd	nd	
	B	nd	2.33	23.1	nd	247	88.2	nd	
Soil	A	0.00	209	nd	nd	3.98	nd	nd	
	B	0.00	1.80	nd	nd	14.6	nd	nd	
Stages I + II + III—day 244^c^									This study
Soil + ditch	na	0.00–0.00 mdn = 0.00	0.00–2.02 mdn = 0.00	25.6–36.8 mdn = 39.8	0.15–48.8 mdn = 39.8	23.5–32.2 mdn = 28.7	40.5–53.6 mdn = 46.9	nd	
Stage IV—day 300^a^									
Soil + ditch	na	235– 538 mdn = 394	7.67–191 mdn = 92.3	nd	nd	nd	nd	275–492 mdn = 334	

*na* not applicable, *nd* not degraded

^a^Initial concentration = 500 µg/L

^b^Initial concentration = 500–1000 µg/L

^c^Initial concentration = 50 µg/L

### Strategies for reactivation of micropollutant biodegradation

#### Addition ofcomplex DOC types (Stage I)

**Fig. 2:**
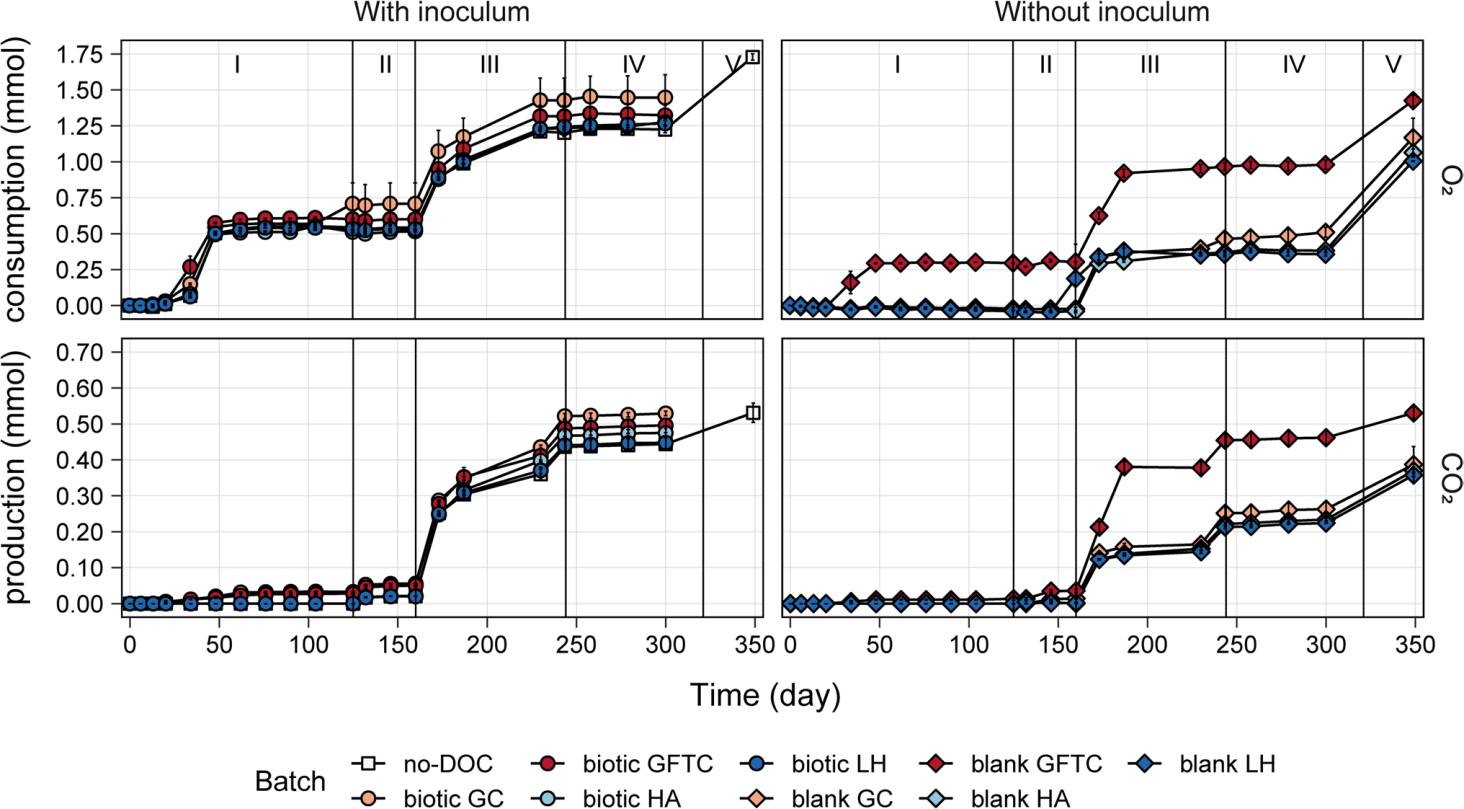
Cumulative O_2_ consumption and CO_2_ production for the no-DOC, biotic, and blank batches across the different stages

During Stage I, the potential of four different types of complex DOC to stimulate micropollutant biodegradation by the soil- and ditch-derived microbial communities (i.e., biotic batches) was also tested. Additionally, batches without inoculum but with DOC addition (i.e., blank batches) were performed to assess the biodegradation activity of the DOC extract. For each DOC type, the changes in the different DOC fractions due to biodegradation were similar in both the biotic and blank batches (Supplementary information, Fig. S1). However, DOC consumption contributed only slightly to O_2_ consumption during Stage I (Fig. 2), as the O_2_ consumption was much higher than what would be expected to result from the amount of DOC present. O_2_ consumption can be attributed mainly to nitrification activity as demonstrated by the fact that (i) the batches for which O_2_ consumption was observed correspond to the batches in which ammonium was consumed (Fig. 2 and S2), (ii) both processes occurred mainly between day 34 and 48, and (iii) the O_2_ amount required for the ammonium consumption observed (0.48 ± 0.10 and 0.43 ± 0.04 for the batches with inoculum and the blank GFTC batch, respectively) are comparable to the O_2_ consumption observed.

Similar to the no-DOC batches, 2,4-D, ANP, and CLZ MDP were the only micropollutants biodegraded in the biotic batches during Stage I (Fig. 1). The lag phase, rate, and extent observed for each micropollutant were similar among all biotic batches but also to the ones observed for the no-DOC batches (“Initial micropollutant biodegradation activity” section). In the biotic batches, like in the no-DOC batches, CLZ MDP was biodegraded at a slower rate and with lower removal efficiencies—26.5 ± 2.5, 39.5 ± 2.6, 23.8 ± 3.0, and 35.3 ± 6.9% (day 125) for the biotic GC, GFTC, HA, and LH batches, respectively. These results show that 2,4-D, ANP, and CLZ MDP biodegradation was not affected (i.e., not stimulated nor inhibited) by DOC addition and its biodegradability. Some authors have reported a positive correlation between the biodegradable DOC (BDOC) amount and micropollutant biodegradation (Lim et al. 2008; Luo et al. 2019). The complex DOC types selected have a high content of humics and hydrophobic organic carbon (69.2–91.25% w/w, Table S3), which are typically recalcitrant DOC fractions, and only a small amount of DOC was added (10 mg-C/L). Therefore, it is likely that the BDOC amount in the batch bottles was not high enough to biostimulate additional micropollutant biodegradation.

Micropollutant biodegradation activity was also observed in batches without inoculum, i.e. blank batches (Fig. 1). Biodegradation of 2,4-D and ANP was observed in the blank GC and blank GFTC batches, respectively. Hence, green and GFT composts contain microorganisms able to biodegrade 2,4-D and ANP, respectively, and these microorganisms are extracted together with the DOC. The presence of microorganisms able to biodegrade 2,4-D under aerobic conditions in GC extract had been previously reported by Aldas-Vargas et al. (2021). In the present study, biodegradation of 2,4-D occurred in only two of the blank GC replicates. The GC extract likely contained a low number of microorganisms, including 2,4-D degraders. Thus, the volume added to each bottle (10 mL) was not enough to capture the full microbial and metabolic diversity of the extract in all bottles, and consequently, 2,4-D degraders were not present in one replicate. In the two 2,4-D degrading replicates, biodegradation occurred at a slower rate than in the bottles with inoculum, with 100% removal only being observed by day 13. This further supports the hypothesis that GC extract contained a lower concentration of 2,4-D degraders than the inoculum. ANP biodegradation in the blank GFTC batches showed a shorter lag phase, of about 20 days, than in the batches with inoculum. However, ANP was biodegraded at a lower rate and to a lower extent. No micropollutant biodegradation was observed in the blank HA and LH batches. The source of DOC used in these batches originated from oligotrophic environments, humic acids from natural groundwater, and liquid humus from leonardite, and therefore, these DOC extracts contained very few microorganisms. Overall, these results show that DOC extracted from natural sources contains micropollutant-degrading microorganisms.

The fact that the ANP was biodegraded only in the batches for which nitrification activity was observed, i.e., no-DOC, all biotic, and blank GFTC batches (Fig. 1 and S2), and that both processes happened at the highest rate simultaneously strongly suggests that ANP biodegradation resulted from autotrophic co-metabolic biodegradation. The aspecific catalytic activity of the ammonia monooxygenase (AMO) enzyme can oxidize a wide range of compounds containing alkyl groups and/or benzene rings (Fernandez-Fontaina et al. 2016; Su et al. 2021), and therefore, nitrifying bacteria may be responsible for the observed ANP biodegradation via co-metabolic biodegradation. For example, Ooi et al. (2018) compared the biodegradation of multiple pharmaceuticals under nitrifying and denitrifying conditions, reporting higher ANP removal rates in all nitrifying bioreactors.

#### Addition of simple DOC (Stage II+III)

Stages II and III aimed to test the effect of simple and easily biodegradable DOC on micropollutant biodegradation. On day 125, a small amount of acetate (4 mg-C/L) was added to all batches—Stage II. The added acetate was fully consumed within 7 days, except for the blank GFTC batch, for which full consumption was observed by day 21 (Fig. S3). Although CLZ MDP was further biodegraded in the batches with inoculum (i.e., no-DOC and biotic batches), no additional micropollutant biodegradation was detected. Hence, on day 160, a much higher amount of acetate (108 mg-C/L) was added to all batches—Stage III. Acetate was fully consumed within 13 days (Fig. S3), resulting in simultaneous O_2_ consumption and CO_2_ production (Fig. 2). The addition of high acetate concentrations stimulated the biodegradation of gabapentin (GAB) and 1H-benzotriazole (1H-BTR).

**Fig. 3:**
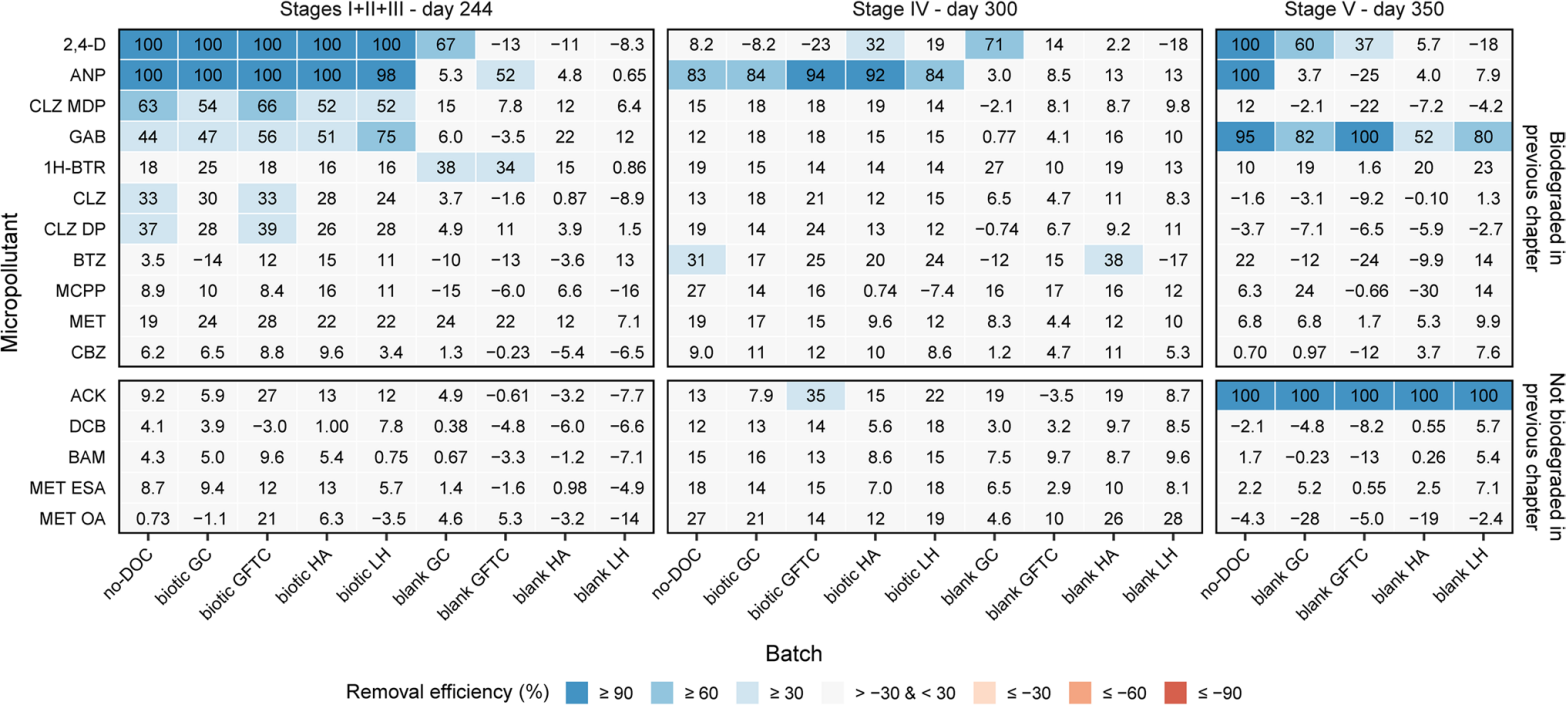
Micropollutant removal efficiencies for the no-DOC, biotic, and blank batches across the different stages

GAB was biodegraded in the batches with inoculum, although not in all replicates (Fig. 1). In our previous research, GAB was biodegraded by the soil and ditch microbial communities only during the 1st spike (Branco et al. 2023). Therefore, GAB degraders subsisted for 4.5 months without the addition of organic carbon sources, but only a very low number was likely present in the inoculum. Consequently, these microorganisms were not inoculated in all bottles, and GAB biodegradation could only be stimulated in some replicates. In the GAB biodegrading replicates, biodegradation occurred between days 125 and 230 with removal efficiencies ranging from 30.8 to 99.8% and threshold concentrations as low as 0.15 µg/L. These concentrations are much lower than the obtained in the previous study (Table 3), suggesting that acetate addition not only stimulated the start of GAB biodegradation but also promoted its biodegradation to a higher extent. On the other hand, 1H-BTR was biodegraded in the blank GC and GFTC batches between days 125 and 230 with removal efficiencies of 38.0 ± 7.2 and 33.5 ± 7.3%, respectively (Fig. 1). Although 1H-BTR removal efficiencies were below 30% by the end of Stage III (Fig. 3), biodegradation of this micropollutant (also biodegraded by ditch and soil in our previous study (Branco et al. 2023)) appears to have started in the batches with inoculum after the addition of 108 mg-C/L of acetate (Fig. 1).

Studies investigating the use of acetate at different concentrations for biostimulation of micropollutant biodegradation suggest that this strategy is successful for a limited number of micropollutants but shows no clear correlation with the concentration of acetate added (Onesios and Bouwer 2012; Abromaitis et al. 2016; Liang et al. 2019). Nevertheless, co-metabolism and growth of micropollutant degraders are pointed out as the mechanism behind improved micropollutant biodegradation (Abromaitis et al. 2016; Liang et al. 2019). GAB and 1H-BTR biodegradation only started when high acetate concentrations were added. Additionally, the removal of these compounds was prolonged after acetate depletion. Therefore, degradation of GAB and 1H-BTR due to the aspecific catalytic activity of enzymes involved in acetate consumption could have occurred. Acetate could have also supported the growth of microorganisms able to degrade the two micropollutants. Furthermore, microbial respiration continued throughout the duration of Stage III even after acetate depletion (Fig. 2), suggesting that the high microbial activity resulting from acetate consumption led to biomass growth and production of organic compounds on which other bacteria can cross-feed (Smith et al. 2019). The use of these biomass and organics as primary substrates could have stimulated GAB and 1H-BTR biodegradation by (i) supporting the growth of the degrading microorganisms, (ii) activating enzymes with unspecific catalytic activity toward micropollutants, or (iii) supplying energy and building blocks for induction of catabolic genes by the degrading microorganisms.

During Stage III, CLZ MDP biodegradation continued in the batches with inoculum. A removal efficiency of 56.6 ± 9.6% was achieved. By the end of Stage III, removal of chloridazon (CLZ) and chloridazon-desphenyl (CLZ DP) was observed in the no-DOC and biotic GFTC (Fig. 3). For the remaining batches with inoculum, CLZ and CLZ MDP removal efficiencies were below 30% by the end of Stage III (Fig. 3). However, biodegradation of these micropollutants appears to have already started in all batches with inoculum on day 34 (Fig. 1), having a similar lag phase as for CLZ MDP, suggesting that DOC addition—both acetate and complex DOC—did not play a role in the biodegradation of CLZ and its metabolites. CLZ and CLZ DP were biodegraded by the soil and ditch microbial communities during the 2nd spike of our previous research (Branco et al. 2023). The microorganisms able to biodegrade these two compounds were, therefore, able to restart biodegradation when micropollutant concentrations increased again but at a much slower rate than in the previous experiment (Table 3). Finally, after the addition of high acetate concentrations, the concentration of ANP, which had been fully removed during Stage I, increased to 2.60 ± 0.46 µg/L in the batches with inoculum (day 187) (Fig. 1). These results show that a small part of ANP had been adsorbed instead of biodegraded and that acetate acted as a desorbent. ANP was again fully removed by the end of Stage III, either due to biodegradation or re-adsorption.

#### Increase in micropollutant concentration (Stage IV)

In order to understand if the low micropollutant concentration (50 µg/L) could be limiting the biodegradation of the persistent micropollutants, the concentration of all micropollutants was increased to approximately 500 µg/L (concentration used in the previous experiment (Branco et al. 2023)) on day 244—Stage IV. This increase resulted in the restart of biodegradation of the previously depleted ANP in all batches with inoculum (i.e., no-DOC and biotic batches) within 14 days of the increase in concentration (removal efficiency = 86.8 ± 8.9%) (Fig. 1). 2,4-D biodegradation also quickly restarted in the blank GC batch, with full removal within 14 days (the replicate that was inactive on 2,4-D during Stage I remained inactive in Stage IV) (Fig. 1). However, among the batches with inoculum, which had previously shown the capacity to fully biodegrade 2,4-D, biodegradation of this micropollutant was only observed in one replicate of the biotic HA batches (removal efficiency = 52.4%) (Fig. 1). DOC concentration and biodegradability have been reported to determine microbial community composition, with higher concentrations and biodegradability being associated with lower community diversity (Li et al. 2012, 2013a). Therefore, it can be inferred that the high acetate concentrations added in the previous stage (III) resulted in the development of a community with low diversity and a high abundance of microorganisms able to degrade acetate. Among the diversity lost were likely 2,4-D degraders, resulting in the loss of 2,4-D biodegradation capacity observed.

Biodegradation of CLZ, CLZ DP, CLZ MDP, and 1H-BTR appears to have continued during Stage IV (Fig. 1), but a high removal extent was not attained due to the low removal rates and short duration of this stage (77 days) (Fig. 3). Finally, the increase in bentazon (BTZ) and acesulfame K (ACK) concentration appears to have induced the biodegradation of these micropollutants in some replicates with and without inoculum (Fig. 1). These results suggest that concentrations higher than 50 µg/L are necessary to induce the metabolization of BTZ and ACK. In fact, BTZ biodegradation in both our previous (Branco et al. 2023) and current studies showed a threshold concentration of 250 µg/L (Table 3), indicating the need for high concentrations to keep its biodegradation active by the microbial community present. Additionally, BTZ was only biodegraded during the 1st spike of our previous experiment, when microbial activity was higher due to a higher concentration of organic carbon and of a more biodegradable nature (with origin in the inoculum) at that point of the study. Therefore, it is possible that the amendment with high acetate concentrations before the increase in micropollutant concentration also positively contributed to the biodegradation of BTZ.

#### Inoculation with activated sludge effect (Stage V)

High microbial diversity and richness have been positively correlated with micropollutant biodegradation (Li et al. 2014; Stadler et al. 2018; Branco et al. 2023). Hence, the final stage (V) consisted of inoculating the no-DOC and blank batches with an activated sludge microbial community (often reported to have high microbial richness and diversity Zhang et al. 2018; Yang et al. 2020; Han et al. 2021)) to explore the possibility of bioaugmentation with this type of microbial community (i.e., no-DOC batches) as well as to understand if the remaining DOC fractions from the complex DOC types would have a positive effect on micropollutant biodegradation by this community (i.e., blank batches). The addition of activated sludge resulted in an increase in microbial respiration (Fig. 2), which likely resulted from the consumption of the DOC still present in the activated sludge by the microorganisms. During Stage V, four micropollutants were biodegraded—2,4-D, ANP, ACK, and GAB (Fig. 1). ACK and GAB were biodegraded in all batches. Since neither ACK nor GAB biodegradation activity had been observed in the previous stages for the blank batches and only GAB was biodegraded in the no-DOC batches, biodegradation of these compounds during Stage V was performed by microorganisms present in the activated sludge. In fact, biodegradation of both compounds by activated sludge has been reported, with biodegradation of ACK appearing to be widespread in WWTPs (Kasprzyk-Hordern et al. 2009; Castronovo et al. 2017; Sun et al. 2022). Full ACK biodegradation occurred in all batches (Fig. 3) and was not affected (i.e., not stimulated nor inhibited) by the complex DOC sources. GAB removal efficiencies varied between 38.3 and 100%. A correlation between lower removal efficiencies and the DOC type could not be established as removal efficiencies differed widely between replicates of the same batch and could, therefore, arise from the inoculation of a lower number of GAB degraders in some bottles.

On the other hand, ANP biodegradation only occurred in the no-DOC batches (removal efficiency = 100%), resulting from the activity of the initial soil- and ditch-derived inoculate that had already been observed in the previous stage (IV) following the increase in micropollutant concentrations (Fig. 1). Lastly, 2,4-D biodegradation varied the most among the batches inoculated with activated sludge (Fig. 1), occurring both in batches that had previously shown 2,4-D degradation activity (i.e., no-DOC) and in batches without previous 2,4-D activity (i.e., one replicate of blank GC and blank GFTC batches). However, 2,4-D also remained undegraded in other batches (i.e., blank HA and LH batches and one replicate of the blank GFTC batches). Therefore, it was not possible to conclude if its biodegradation was performed by the activated sludge community and inhibited by some DOC fractions present and/or if the microorganisms performing 2,4-D biodegradation were already present in some of the batch bottles and their activity was stimulated by compounds, such as DOC, present in the activated sludge inoculum.

### Insights on stimulating (reactivation of) micropollutant biodegradation

The results obtained in this study give insights into how reactivation of micropollutant biodegradation can be stimulated after a prolonged incubation period without substrates—either micropollutants or other organic carbon sources—as well as on stimulation of micropollutant biodegradation in general. No biodegradation was observed for 7 of the 16 micropollutants studied, even when DOC was added, micropollutant concentration increased, or bioaugmentation with a diverse microbial community. Of these, MCPP, metolachlor (MET), and carbamazepine (CBZ) were biodegraded by the soil microbial community (from which the inoculum was derived) under aerobic conditions during the 2nd spike of our previous study (Fig. 3) (Branco et al. 2023). Therefore, the 2-month period without substrate addition (i.e., micropollutants and other organic carbon sources) resulted in the permanent loss of micropollutant biodegradation capacity by the community. The prolonged period with lower MCPP, MET, and CBZ as well as lower biodegradable organic carbon concentrations likely resulted in a big decrease and possible extinction, of the bacteria able to biodegrade these compounds (Nguyen et al. 2021). Consequently, biodegradation activity could not be recovered even when the micropollutant concentration was increased to 500 µg/L or different DOC types were amended. Therefore, periods of no/decreased substrate availability in the environment can result in permanent loss of micropollutant biodegradation capacity within a microbial community. Furthermore, these results also show that amendment with DOC and high micropollutant concentrations were not enough to stimulate the degradation of dichlobenil, BAM, metolachlor ESA and OA, and acesulfame K by the combined soil and ditch microbial community, suggesting that the potential (i.e., catabolic genes) for biodegradation of such micropollutants is also absent in this community. Finally, is worth noting that recent studies, including our previous study with the same soil microbial community, have reported higher MET biodegradation under anaerobic conditions compared with aerobic conditions (Elsayed et al. 2015; Kanissery et al. 2018, 2021; Branco et al. 2023). It is, therefore, possible that the redox condition chosen, i.e., aerobic, affected the results observed concerning MET biodegradation.

A possible solution to promote micropollutant biodegradation in an environment whose microbial community lacks the genetic potential for such a process is bioaugmentation. In this technique, a non-native microbial community or strain with micropollutant degradation capacity is added to the contaminated environment (Helbling 2015; Cycoń et al. 2017). It is therefore important to find a source of microorganisms with a capacity to biodegrade the micropollutant(s) of interest. Activated sludge appears as a promising source of such microorganisms, having shown the capacity to degrade acesulfame K, gabapentin, and, possibly, 2,4-D (Stage V). This capacity has been previously described for activated sludge originating from other WWTPs (Branco et al. 2023). Our results also show that DOC extracted from natural sources, in particular with origin in non-oligotrophic environments, can be a source of micropollutant-degrading microorganisms and, possibly, be used as inoculum for bioaugmentation (Stages I and III). Nonetheless, the application of bioaugmentation techniques in natural environmental systems is still limited, in particular by the ability of the native microbial community, which is adapted to the conditions of the bioremediated environmental compartment, to outcompete the exogenous community (Helbling 2015; Cycoń et al. 2017).

On the other hand, the combined soil and ditch microbial community retained the capacity to degrade 7 micropollutants (Stages I to IV)—2,4-D, bentazon (BTZ), chloridazon (CLZ), chloridazon-desphenyl (CLZ DP), chloridazon-methyl-desphenyl (CLZ MDP), gabapentin (GAB), and antipyrine (ANP) (Fig. 3). 2,4-D, ANP, CLZ, CLZ DP, and CLZ MDP biodegradation restarted when these micropollutants were present again at low concentrations without a biostimulation strategy being applied, while reactivation of GAB required amendment with high concentration of acetate and reactivation of BTZ in some batch bottles occurred when its concentration increased to 500 µg/L. Biodegradation of some of these compounds was slower than in our previous study, likely due to the lower amount of micropollutant degraders present in the community after the prolonged period without substrate addition. However, threshold concentrations were similar to before the period without substrate addition. These results show that microbial communities are able to maintain most of their micropollutant biodegradation capacity and reactivate it as soon as micropollutants are available again, although slower rates may be observed. It is worth highlighting that high threshold concentrations of 2.33–394 µg/L were found in both our previous and present studies (Table 3), with most micropollutants not having been fully biodegraded even after prolonged experimental periods (175 and 349 days, respectively). Such limitation stresses the urgency to find new ways to stimulate micropollutant biodegradation in different environmental compartments.

Our microcosm studies demonstrated that the addition of high concentrations of an easily biodegradable DOC (Stage III) but also of ammonium (ANP biodegradation during Stage I) is a feasible strategy to reactivate micropollutant biodegradation, which could also be used for stimulation of micropollutant biodegradation in general. In contrast, complex DOC sources like the ones obtained from compost, groundwater humics, and leonardite appear to have limited potential for the stimulation of micropollutant biodegradation due to their recalcitrance. Even though promoting biodegradation of some micropollutants, the amendment with high concentrations of acetate resulted in the loss of 2,4-D biodegradation capacity by changing microbial community composition. Hence, the amendment of contaminated sites with easily biodegradable DOC sources in high concentrations must be performed with care, and its effect on the biodegradation of all micropollutants present must be investigated beforehand. Finally, albeit not a biostimulation strategy, it is possible that, as a result of contamination, the accumulation of a micropollutant in the environment and consequent increase in concentration can eventually lead to the reactivation of micropollutant biodegradation (Stage IV).

## Conclusions

This study aimed to better understand how micropollutant biodegradation activity develops after a prolonged period of no substrate addition (i.e., micropollutants and other organic carbon sources) and how possible losses in such activity can be mitigated. This study shows that:

- Although the biodegradation capacity of 3 micropollutants—MCPP, MET, and CBZ—was permanently lost after a period of 2 months without micropollutant and other organic carbon addition, biodegradation activity could be restored for most micropollutants studied.
- The biodegradation activity of 2,4-D, ANP, CLZ, CLZ DP, and CLZ MDP restarted when these micropollutants were again present. For some of these compounds, biodegradation rates were slower than when the microbial community was initially exposed to these micropollutants (in our previous study), but threshold concentrations similar to before the period without substrate addition were achieved.
- ANP biodegradation strongly correlates with nitrification, suggesting that AMO activity could be responsible for the co-metabolic biodegradation of this micropollutant.
- GAB biodegradation activity was regained after amendment with a high concentration of acetate (easily biodegradable DOC). Acetate directly supported the growth of GAB degraders, or the biomass/organics resulting from the high microbial activity supported by acetate consumption were used as primary substrates for (i) growth of the degrading microorganisms, (ii) activation of enzymes with unspecific catalytic activity toward these micropollutants, or (iii) energy and building blocks for induction of catabolic genes by the degrading microorganisms.
- An increase of BTZ concentration to 500 µg/L was necessary for the biodegradation of these compounds to be reactivated.

Additionally, the results also showed that amendment with high concentrations of easily biodegradable substrates can result in the loss of biodegradation capacity of other micropollutants. Hence, when applying this type of amendment, its effect on the biodegradation of all micropollutants present must be considered. While the addition of complex DOC types had no effect on micropollutant biodegradation, this DOC extracted from natural sources presented the capacity to biodegrade micropollutants, offering the possibility to be used as inoculum for bioaugmentation. Activated sludge could also be a promising source of microbial communities for bioaugmentation. More research is, however, needed in this field. These are key insights on the capacity of a microbial community to be reactivated for micropollutant biodegradation, for example, when micropollutants are available again in a system with fluctuating micropollutant concentrations, such as WWTPs, constructed wetlands, and activated carbon and rapid sand filters.

### Supplementary Information

Below is the link to the electronic supplementary material.

Supplementary file1 (DOCX 33686 KB)

## Data Availability

The link to the data and code is shared in the materials and methods section of the manuscript.
