# Supplementary material for: Exploring long-term retention and reactivation of micropollutant biodegradation capacity
Source: Environ Sci Pollut Res Int. 2024 Jul 10;31(34):47055–70. doi: 10.1007/s11356-024-34186-w (PMC11296967; doi:10.1007/s11356-024-34186-w)
Supplement: Supplementary file 1 — Supplementary file1 (DOCX 33686 KB) [file 11356_2024_34186_MOESM1_ESM.docx]

**Supplementary information**

*Exploring long-term retention and reactivation of micropollutant biodegradation capacity*

Rita H.R. Branco^a,b^, Roel J.W. Meulepas^b^, Huub H.M. Rijnaarts^a^, Nora B. Sutton^a^*

^a^ Environmental Technology, Wageningen University & Research, P.O. Box 47, 6700 AA Wageningen, the Netherlands

^b^ Wetsus, European Centre of Excellence for Sustainable Water Technology, P.O. Box 1113, 8900 CC Leeuwarden, the Netherlands

* Corresponding author: nora.sutton@wur.nl

**Tables**

**Table S1.** Concentration and frequency of detection of the micropollutants studied in Dutch groundwater

(a) Pesticides

| **Compound** | **Year** | **Screen depth (m below soil surface)** | **# sampling sites** | **# total meas.** | **< LOD/LOQ/LOR** | | **≤ norm^a^** | | | **> norm^a^** | | | **Ref.** |
| --- | --- | --- | --- | --- | --- | --- | --- | --- | --- | --- | --- | --- | --- |
|  |  |  |  |  | **# meas.** | **range (µg/L)** | **# meas.** | **range (µg/L)** | **mean (µg/L)** | **# meas.** | **range (µg/L)** | **mean (µg/L)** |  |
| 2,4-D | 2011-2021 | 0.22-200.12 | 1058 | 4056 | 4011 | 0.01-0.50 | 44 | 0.01-0.02 | 0.02 | 1 | 0.29 | NA | (Wageningen University and Research, 2022) |
| MCPP | 2011-2021 | 0.22-276.00 | 1036 | 3839 | 3447 | 0.01-0.20 | 300 | 0.01-0.10 | 0.04 | 92 | 0.11-6.16 | 0.22 |  |
| CLZ | 2011-2021 | 0.22-200.12 | 1044 | 4138 | 3932 | 0.01-0.20 | 190 | 0.01-0.10 | 0.01 | 16 | 0.11-4.09 | 0.25 |  |
| DCB | 2011-2021 | 0.22-200.12 | 870 | 3419 | 3362 | 0.02-0.15 | 50 | 0.02-0.07 | 0.03 | 7 | 0.11-0.36 | 0.15 |  |
| BAM | 2011-2021 | 0.22-276.00 | 1020 | 4105 | 3469 | 0.01-0.60 | 316 | 0.01-0.10 | 0.04 | 320 | 0.11-6.40 | 0.30 |  |
| BTZ | 2011-2021 | 0.22-276.00 | 1067 | 4300 | 3449 | 0.01-0.30 | 625 | 0.01-0.10 | 0.03 | 226 | 0.11-15.00 | 0.26 |  |
| CLZ DP | 2013-2021 | 0.22-188.22 | 714 | 1867 | 1126 | 0.02-0.50 | 272 | 0.02-0.10 | 0.05 | 469 | 0.11-24.00 | 0.59 |  |
| CLZ MDP | 2013-2021 | 0.22-188.22 | 714 | 1867 | 1519 | 0.01-0.20 | 166 | 0.01-0.10 | 0.04 | 182 | 0.11-3.80 | 0.42 |  |
| MET ESA | 2009-2020 | 0.98-180.00 | 202 | 773 | 462 | 0.01-0.05 | 136 | 0.01-0.10 | 0.04 | 175 | 0.11-21.00 | 0.45 |  |
| MET OA | 2009-2021 | 0.98-188.22 | 248 | 988 | 758 | 0.01-0.05 | 70 | 0.01-0.10 | 0.04 | 160 | 0.11-19.00 | 0.39 |  |

(b) Pharmaceuticals, industrial substances, and artificial sweeteners

| **Compound** | **Year** | **Screen depth (m below soil surface)** | **# sampling sites** | **# total meas.** | **< LOD/LOQ/LOR** | | **≥ LOD/LOQ/LOR** | | | **Ref.** |
| --- | --- | --- | --- | --- | --- | --- | --- | --- | --- | --- |
|  |  |  |  |  | **# meas.** | **range (µg/L)** | **# meas.** | **range (µg/L)** | **mean (µg/L)** |  |
| ANP | 2015-2016 and  2018-2019 | *na* | *na* | 631 | 588 | 0.01 | 43 | 0.01-0.47 | 0.06 | (van Loon et al., 2020) |
| CBZ |  | *na* | *na* | 631 | 595 | 0.01 | 36 | 0.01-0.59 | 0.06 |  |
| GAB | 2021 | ≤ 16 | 45 | 61 | 54 | *na* | 7 | 0.06-0.47 | *na* | (Provincie Overijssel, 2023) |
| ACK | 2021 | ≤ 16 | 45 | 61 | 27 | *na* | 34 | 0.01-3.11 | *na* |  |
| 1H-BTR | *na* | *na* | *na* | *na* | *na* | *na* | *na* | *na* | *na* | *na* |

# meas. – number of measurements; LOD – limit of detection; LOQ – limit of quantification; LOR – reporting limit; ^a^ norm = 0.01 µg/L

**Table S2** Micropollutants properties. (Pérez-Lucas et al., 2019; ChemSpider, 2023; PPDB, 2023; PubChem, 2023)

| **Compound** | **Water solubility at 20 °C (mg/L)** | **Log K_ow_** | **K_oc_**  **(mobility class)** | **pKa**  **at 25 °C** | **Aqueous**  **stability** | **Half-life in soil (days)** | **Persistence** |
| --- | --- | --- | --- | --- | --- | --- | --- |
| 2,4-D | 24300 | -0.82 | 39.3  (mobile) | 3.40 | Stable | 4.4 | nonpersistent |
| MCPP | 250000 | -0.19 | 47.0  (mobile) | 3.11 | Stable | 8.2 | nonpersistent |
| CLZ | 422 | 1.19 | 120  (moderately mobile) | 3.38 | Stable | 31 | moderately persistent |
| CLZ DP | 87820^a^ | -1.59 | 85  (moderately mobile) | *Na* | *na* | 108 | persistent |
| CLZ MDP | 50240^a^ | -1.38 | 52.07  (mobile) | *Na* | *na* | 145 | persistent |
| DCB | 21.2 | 2.7 | 257  (moderately mobile) | *Na* | Stable | 70 | moderately persistent |
| BAM | 1830 | 0.38 | 30  (mobile) | *Na* | Stable | 137.7 | persistent |
| MET | 530 | 3.4 | 120  (moderately mobile) | *Na* | Stable | 90 | moderately persistent |
| MET ESA | 212461 | -1.89 | 9  (very mobile) | *Na* | *na* | 400 | very persistent |
| MET OA | 360000 | *na* | 17.0  (mobile) | *Na* | *na* | 325 | persistent |
| BTZ | 500 | -0.46 | 55.3  (mobile) | 3.51 | Stable | 20 | nonpersistent |
| ANP | 51900^a^ | 0.38 | 223.1  (moderately mobile) | 1.4 | Stable | 30 | moderately persistent |
| CBZ | 18^a^ | 2.45 | 3871  (slightly mobile) | -3.8, 15.96 | *na* | 75 | moderately persistent |
| GAB | 4490^a^ | -1.10 | 58.96  (mobile) | 3.68, 10.70 | *na* | 30 | moderately persistent |
| ACK | *na* | *na* | *na* | *na* | *na* | *na* | *na* |
| 1H-BTR | 19800^a^ | 1.44 | 996.2  (slightly mobile) | 8.36 | Stable | 30 | moderately persistent |

K_ow_ - octanol-water partition coefficient; K_oc_ - organic carbon normalized sorption coefficient; pKa - acid dissociation constant; *na* – not available; ^a^ water solubility at 25 °C

**Table S3** DOC type composition.

| **DOC type** | **DOC fraction (% w/w)** | | | | |
| --- | --- | --- | --- | --- | --- |
|  | **Biopolymers** | **Humics** | **LMW neutrals** | **LMW acids** | **HOC** |
| GC | 3.74 | 72.3 | 11.2 | 5.76 | 6.90 |
| GFTC | 12.4 | 65.0 | 10.8 | 4.50 | 7.24 |
| HA | 0.00 | 86.0 | 6.14 | 2.54 | 5.25 |
| LH | 9.77 | 39.2 | 10.1 | 4.50 | 30.0 |

LMW – low molecular weight; HOC - hydrophobic organic carbon (DOC fraction remaining in the column).

**Table S4** Final media composition.

| **Compound** | **Conc. final media (mg/L)** |
| --- | --- |
| **Macronutrients** |  |
| NH_4_Cl | 822 |
| MgSO_4_ | 21.3 |
| CaCl_2_.2H_2_O | 38.7 |
| **Micronutrients** |  |
| FeCl_2_.4H_2_O | 0.967 |
| CoCl_2_.6H_2_O | 0.967 |
| MnCl_2_.4H_2_O | 0.242 |
| NiCl_2_.6H_2_O | 0.024 |
| ZnCl_2_ | 0.024 |
| Na_2_SeO_3_.5H_2_O | 0.048 |
| CuCl_2_.2H_2_O | 0.015 |
| HBO_3_ | 0.024 |
| (NH_4_)_6_Mo_7_O_24_.4H_2_O | 0.044 |
| **Vitamins** |  |
| Biotin (vitamin H) | 0.016 |
| p-aminobenzoate (Na-salt) | 0.040 |
| Pantothenate (Na-salt) | 0.040 |
| Pyridoxine (vitamin B6) | 0.080 |
| Nicotinamide | 0.040 |
| Thiamine HCl (vitamin B1) | 0.040 |
| Riboflavine (vitamin B2) | 0.040 |
| Cyanocobalamine (vitamin B12) | 0.001 |
| Folic acid (dihydrate) | 0.016 |
| Lipoic acid (thioctic acid) | 0.040 |
| **Buffer** |  |
| Na_2_HPO_4_.2H_2_O | 869 |
| KH_2_PO_4_ | 316 |
| **Redox indicator** |  |
| Resazurin | 0.242 |
| **Chelating agent** |  |
| EDTA | 0.484 |

**Table S5** Chemical analysis detection limits.

| **Compound** | **Detection limit** |
| --- | --- |
| MCPP, BTZ | 2.0 µg/L |
| 2,4-D, MET ESA | 1.0 µg/L |
| ACK | 0.5 µg/L |
| CBZ, CLZ DP, DCB, ANP, BAM, | 0.2 µg/L |
| GAB, 1H-BTR, CLZ, CLZ MDP, MET, MET OA | 0.1 µg/L |
| Organic carbon | 50 µg/L |
| Acetate | 1.5 mg/L |
| NH_4_^+^ | 38 mg/L |
| NO_3_^-^ | 9 mg/L |
| O_2_ | 0.75 % (v/v) |
| CO_2_ | 0.10 % (v/v) |

**Table S6** Compounds analysed for each batch type.

| **Compound** | **Batch** | | | |
| --- | --- | --- | --- | --- |
|  | **No DOM** | **Biotic** | **Blank** | **Abiotic** |
| Micropollutants | X | X | X | X |
| NH_4_^+^ | X | X | X |  |
| NO_3_^-^ | X | X | X |  |
| Organic carbon | X | X | X |  |
| Acetate | X | X | X | X |
| O_2_ | X | X | X | X |
| CO_2_ | X | X | X | X |

**Table S7** LC-MS standards composition.

| **Standard** | **Compound** | **µg/L** |
| --- | --- | --- |
| **Micropollutants standard** | 2,4-D | 9.52 |
|  | Acesulfame K | 9.52 |
|  | Antipyrine | 9.52 |
|  | BAM | 9.52 |
|  | Bentazon | 9.52 |
|  | Benzotriazole | 9.52 |
|  | Carbamazepine | 9.52 |
|  | Chloridazon | 9.52 |
|  | Chloridazon-desphenyl | 9.52 |
|  | Chloridazon-methyl-desphenyl | 9.52 |
|  | Dichlobenil | 9.52 |
|  | Diclofenac | 9.52 |
|  | Gabapentin | 9.52 |
|  | Metolachlor | 9.52 |
|  | Metolachlor ESA | 9.52 |
|  | Metolachlor OA | 9.52 |
|  | Mecoprop | 9.52 |
| **Internal standard** | Atenolol-D7 | 26.19 |
|  | Ciprofloxacin-D8 | 26.36 |
|  | Dihydrocarbamazepine | 26.20 |
|  | Bentazon-d7 | 25.22 |
|  | Fenoprofen | 26.09 |
|  | Trimethoprim-D9 | 26.01 |

**Table S8** DOC fractions analysed with LC-OCD.

| **Fraction** | **Molecular weight** | **Compounds^a^** |
| --- | --- | --- |
| Biopolymers | > 40 kDa | polysaccharides, proteins and aminosugars |
| Humics | 500 Da – 40 kDa | humic and fulvic acids and their breakdown products |
| LMW acids | < 350 Da | protic organic acids |
| LMW neutrals | < 350 Da | LMW alcohols, aldehydes, ketones, sugars and amino acids |
| HOC^b^ | *na* | long chain aliphatic and polycyclic aromatic material |

LMW – low molecular weight; HOC - hydrophobic organic carbon (DOC fraction remaining in the column); ^a^ (Huber et al., 2011; Rutlidge et al., 2015)

**Figures**

**
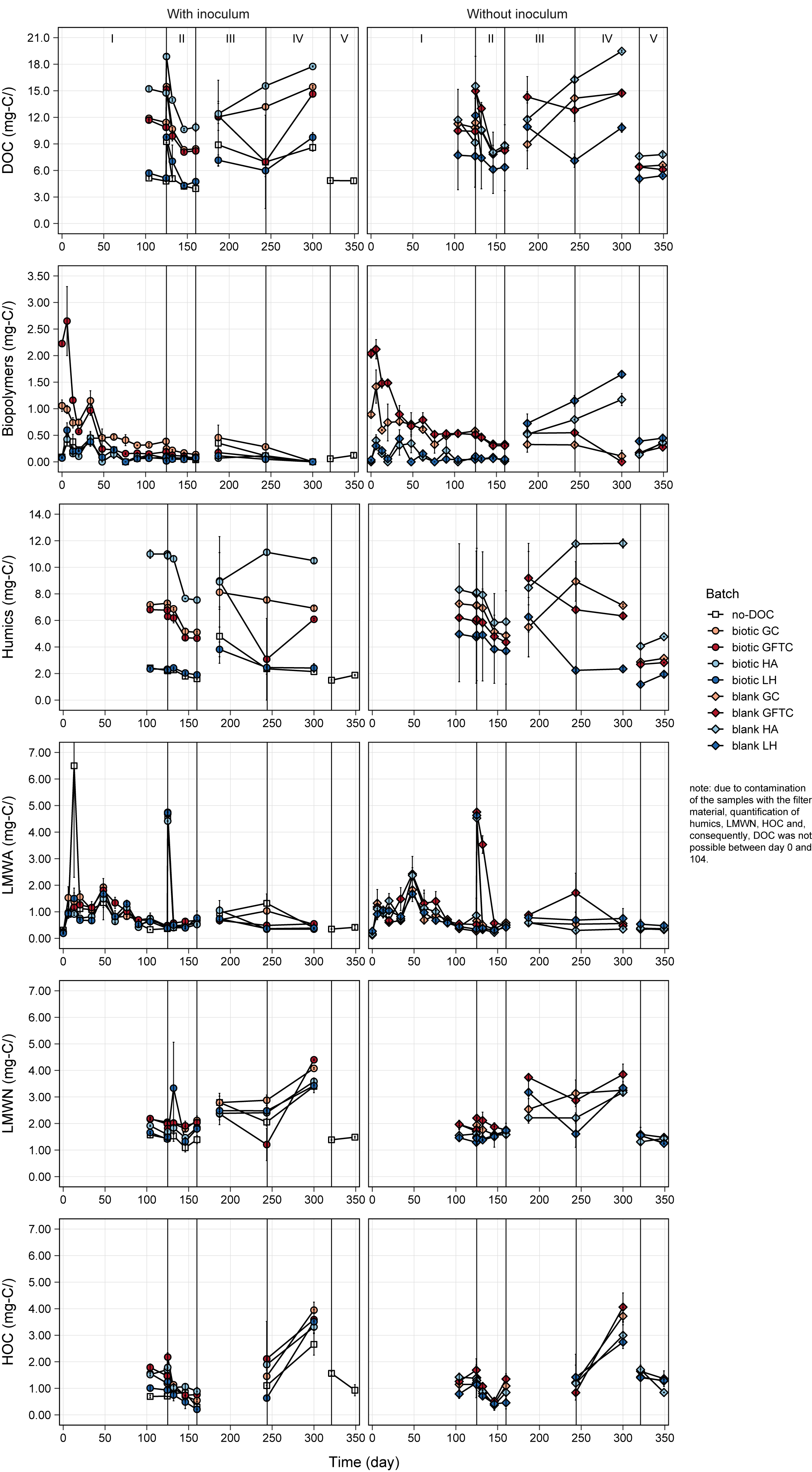
Fig. S1** Total DOC and DOC fractions in time for no-DOC, biotic and blank batches across the different stages.

**
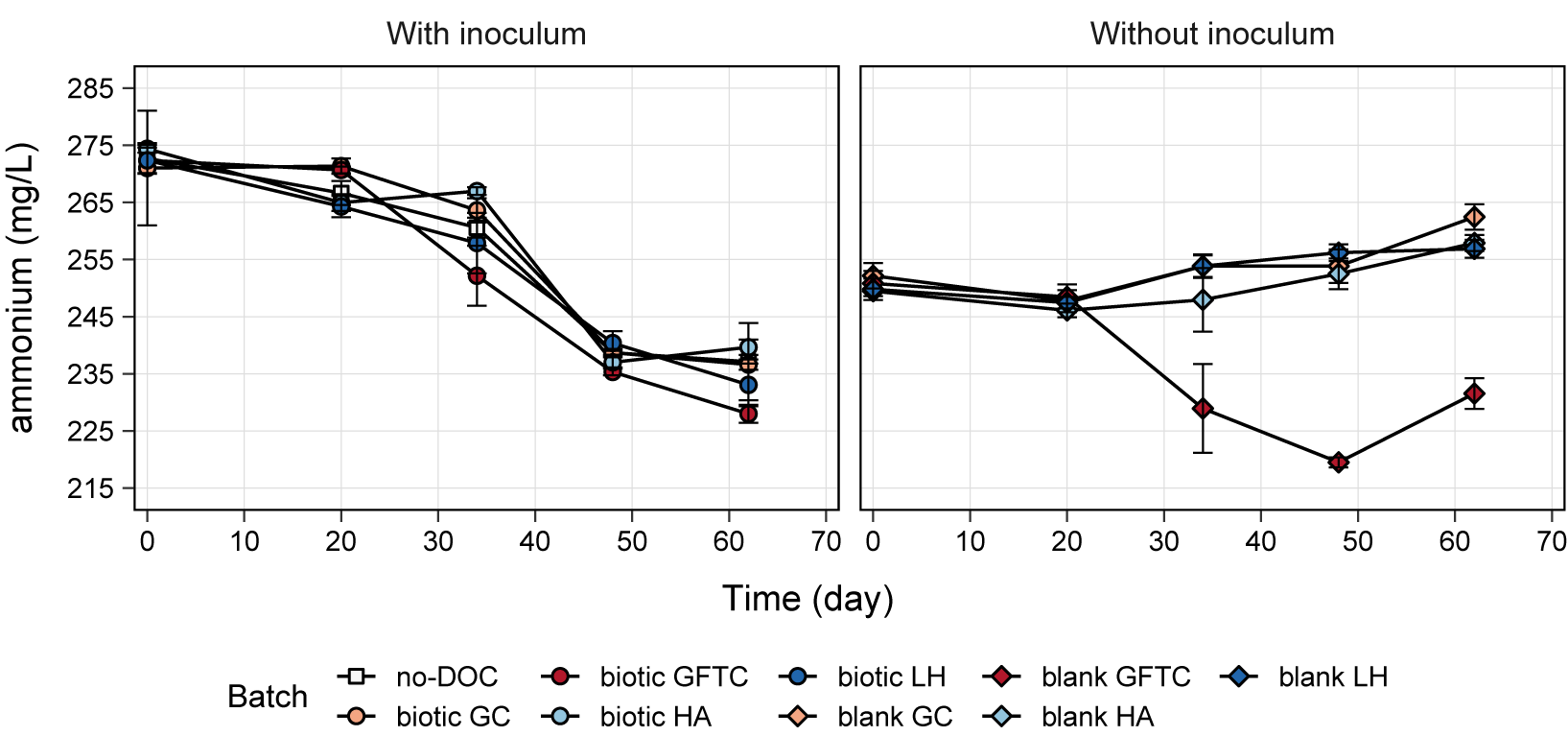
Fig. S2** Ammonium consumption for no-DOC, biotic and blank batches during Stage I.

**
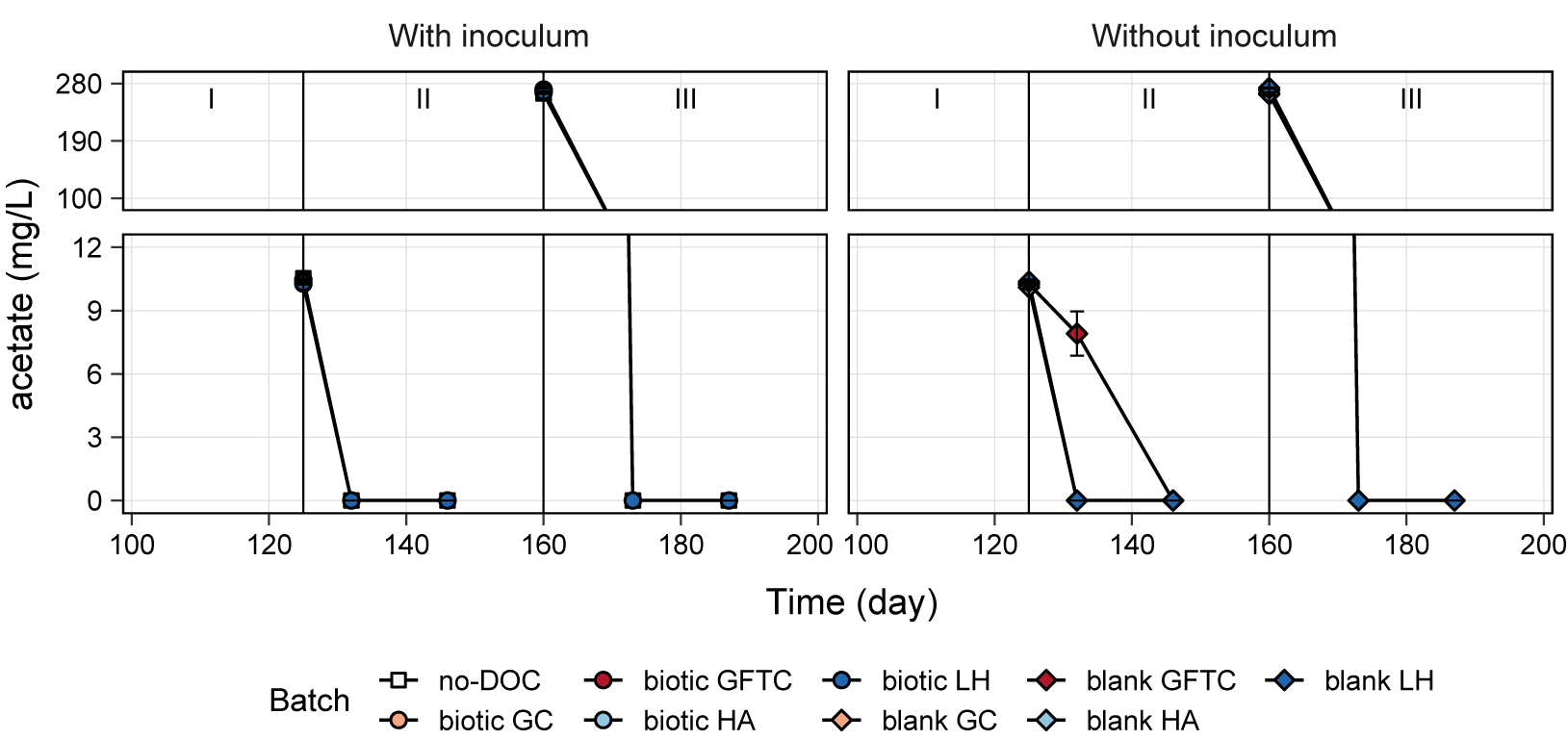
Fig. S3** Acetate consumption for no-DOC, biotic and blank batches during Stages II and III.

**References**

ChemSpider (2023). ChemSpider. *ChemSpider*. Available at: http://www.chemspider.com/

Huber, S. A., Balz, A., Abert, M., and Pronk, W. (2011). Characterisation of aquatic humic and non-humic matter with size-exclusion chromatography – organic carbon detection – organic nitrogen detection (LC-OCD-OND). *Water Research* 45, 879–885. doi: 10.1016/j.watres.2010.09.023

Pérez-Lucas, G., Vela, N., El Aatik, A., and Navarro, S. (2019). “Environmental risk of groundwater pollution by pesticide leaching through the soil profile,” in *Pesticides - Use and Misuse and Their Impact in the Environment*, eds. M. Larramendy and S. Soloneski (IntechOpen). doi: 10.5772/intechopen.82418

PPDB (2023). PPDB. *Pesticide Properties DataBase*. Available at: http://sitem.herts.ac.uk/aeru/ppdb/

Provincie Overijssel (2023). Medicines in the groundwater of Overijssel. *KennisHub Overijssel*. Available at: https://www.kennishuboverijssel.nl/pages/geneesmiddelen-in-het-grondwater-van-overijssel

PubChem (2023). PubChem. *PubChem*. Available at: https://pubchem.ncbi.nlm.nih.gov/

Rutlidge, H., Andersen, M. S., Baker, A., Chinu, K. J., Cuthbert, M. O., Jex, C. N., et al. (2015). Organic characterisation of cave drip water by LC-OCD and fluorescence analysis. *Geochimica et Cosmochimica Acta* 166, 15–28. doi: 10.1016/j.gca.2015.05.042

van Loon, A., Pronk, T., Raterman, B., and Ros, S. (2020). Grondwaterkwaliteit Nederland 2020 - Anorganische parameters, bestrijdingsmiddelen, farmaceutica en overige verontreinigende stoffen in de grondwatermeetnetten van de provincies. the Netherlands: KWR. Available at: https://edepot.wur.nl/531769

Wageningen University and Research (2022). Groundwater Atlas for pesticides in The Netherlands. Available at: https://www.pesticidemodels.eu/groundwateratlas/home
